# Chemically induced mouse liver tumors are resistant to treatment with atorvastatin

**DOI:** 10.1186/1471-2407-14-766

**Published:** 2014-10-15

**Authors:** Albert Braeuning, Philip Bucher, Ute Hofmann, Albrecht Buchmann, Michael Schwarz

**Affiliations:** Institute of Experimental and Clinical Pharmacology and Toxicology, Department of Toxicology, University of Tuebingen, Wilhelmstr. 56, Tuebingen, 72074 Germany; Dr. Margarete Fischer-Bosch Institute of Clinical Pharmacology, Auerbachstr. 112, Stuttgart, 70376 Germany

**Keywords:** Chemical carcinogenesis, HCC, N-nitrosodiethylamine, Ha-ras, Hypolipidemic drug, Oatp1

## Abstract

**Background:**

Atorvastatin is a potent inhibitor of the mevalonate pathway and widely used as a hypolipidemic drug. Some epidemiological studies and animal experiments indicate that the long-term use of atorvastatin and structurally related drugs might be associated with a reduced risk of developing hepatocellular carcinoma (HCC), the most common hepatocellular malignancy in humans. However, the potential of atorvastatin to inhibit HCC formation is controversially discussed.

**Methods:**

Hepatocellular tumors were chemically induced by treatment of C3H/He mice with 10 μg/g body weight N-nitrosodiethylamine and the ability of atorvastatin to interfere with tumor formation was investigated by treatment of mice with 0.1% atorvastatin in the diet for 6 months. Tumor size and tumor multiplicity were analyzed, as were tissue levels of cholesterol and atorvastatin.

**Results:**

Atorvastatin treatment efficiently reduced serum cholesterol levels. However, the growth of tumors driven by activated MAPK (mitogen-activated protein kinase) signaling was not attenuated by the presence of the drug, as evidenced by a lack of reduction of tumor volume or tumor multiplicity by atorvastatin. Levels of the atorvastatin uptake transporters Oatp1a4 and Oatp1b2 were down-regulated at the mRNA and protein levels in chemically induced mouse liver tumors, but without striking effects on atorvastatin concentrations in the tumor tissue.

**Conclusion:**

In summary, the present data provide substantial evidence that atorvastatin does not beneficially influence tumor growth in mouse liver and thereby challenge the hypothesis that statin use might protect against hepatocellular cancer.

**Electronic supplementary material:**

The online version of this article (doi:10.1186/1471-2407-14-766) contains supplementary material, which is available to authorized users.

## Background

Statins are an important and widely used class of hypolipidemic drugs. Their pharmacological efficacy is based on their ability to competitively inhibit 3-hydroxy-3-methyl-glutaryl-CoA reductase (HMGCR), an important and rate-limiting enzyme in the isoprenoid- and cholesterol-synthesizing mevalonate pathway. Apart from their lipid-lowering properties, several epidemiological studies evidence that the long-term use of statins in humans might be causally linked to a reduced risk of developing different types of cancer. For recent review articles about this topic see for example [1–4]. However, there are also studies available which negate the beneficial effects of statin use on cancer incidence or mortality [5, 6]. Another meta-analysis of published data came to the conclusion that there is only weak, inconclusive evidence for a beneficial effect of statin use regarding cancer development [7].

One organ, for which anti-cancer effects of statins have been discussed quite often, is the liver. A number of epidemiological studies and meta-analyses have been published linking statin treatment to a diminished risk for hepatocellular carcinoma (HCC) [8–15]. However, epidemiological evidence for the inhibition of HCC formation by statins is controversial, due to potential confounders, bias, controversies about study design, mechanistic issues, and absence of a duration-risk relationship [10, 16–20]. *In vitro*, different statins are able to inhibit the growth and to induce apoptosis and cell cycle arrest of hepatoma cell lines [21–23]. Atorvastatin was shown to inhibit the growth of HUH-7-derived xenograft tumors [24]. Several genetic or chemical experimental HCC models have been used to analyze potential tumor-inhibiting properties of statins in rodents: transgenic, MYC-driven HCC development was diminished by treatment with atorvastatin [25], as rosuvastatin did in mice developing HCC due to transgenic SV40-T antigen expression [26]. Diethylnitrosamine (DEN)-induced liver tumors in obese mice were suppressed by treatment with pitavastatin [27], and similar effects were observed in DEN-treated rats when lovastatin was co-administered [28]. By contrast, no tumor-inhibitory effect of atorvastatin was detectable on the development of murine TSC2-associated liver hemangiosarcomas [29].

In consideration of ambiguous epidemiological data and of the fact that different statins produce highly divergent effects on hepatoma cells [30], further animal experiments with different statins and HCC models are needed. Therefore, in the present study, we analyzed potential protective effects of atorvastatin treatment on the growth of chemically induced mouse liver tumors harboring an activated Ras/Raf/MAPK (mitogen-activated protein kinase) signaling pathway. This type of tumors was chosen because the MAPK is frequently overactivated in human HCC [31] and because previous studies by our group revealed that enzymes of cholesterol biosynthesis, namely *Hmgcs1* (3-hydroxy-3-methylglutaryl-CoA synthase) and *Lss* (lanosterol synthase), are transcriptionally up-regulated in chemically induced mouse liver tumors with an activated Ras/Raf/MAPK pathway. In addition, a down-regulation of *Cyp7a1*, encoding the rate-limiting enzyme in the cholesterol-metabolizing bile acid synthesis pathway, was observed in these tumors and accordingly, tumors with activated MAPK signaling contained significantly higher levels of cholesterol [32, 33]. Assuming the observed changes in the tumor’s metabolic profile being advantageous for its growth or survival, inhibitors of cholesterol biosynthesis might be especially suited as a treatment option for this particular liver tumor phenotype.

## Methods

### Animal experiment

Male inbred C3H/HeN mice (Janvier Labs, Saint-Berthevin, France) were injected with a single i.p. dose of 10 μg/g body weight of the genotoxic tumor inducer DEN (dissolved in 0.9% NaCl) at 12–14 days of age. In the course of hepatic DEN metabolism, ethyl cations are formed which form covalent adducts with the DNA, thus giving rise to gene mutations. This procedure follows an established protocol as used e.g. in [32–34], and the resulting tumor phenotype has been extensively studied [33, 34]. One week later, animals were stratified into two groups: one group (25 mice) received standard control diet (Ssniff, Soest, Germany), the second group (24 mice) was fed a modified diet (Ssniff) containing 0.1% wt/wt atorvastatin (Ca^2+^ salt, catalog no. A2476; TCI, Tokyo, Japan). Atorvastatin was chosen because it is the most commonly prescribed and also one of the most potent statins on the market. The selected concentration of the drug has proven to be efficacious to reduce cholesterol levels in mice without exerting toxic effects [29]. Mice had access to food and tap water *ad libitum* and were kept on a 12 h dark/light cycle. After 6 months of continuous atorvastatin treatment, the mice were killed; livers were excised and immediately frozen on dry ice for immunohistochemistry. Aliquots of livers and serum samples to be used for cholesterol determination were snap-frozen in liquid nitrogen. All animals received humane care and protocols complied with institutional guidelines. Ethical approval for the animal study was obtained from the Regierungspräsidium Tübingen (permission no. TO6/10).

### Immunohistochemical staining

Cryostat sections (10 μm thickness) were fixed in 4% paraformaldehyde and stained with hematoxylin/eosin or immunohistochemically for glutamine synthetase, E-cadherin, and phosphorylated ERK1/2 (extracellular signal-regulated kinase) using the antibodies and methodology described in previous papers [33, 35]. For staining of OATP1A4 and OATP1B2, primary antibodies against the two transporters (Santa Cruz Biotechnology, Santa Cruz, CA, USA; catalog no. sc-47270 and sc-18436) were used at 1:50 dilution in combination with horseradish peroxidase-conjugated donkey-anti-goat secondary antibodies (1:50 dilution; Santa Cruz Biotechnology; catalog no. sc-3851) and the substrates 3-amino-9-ethylcarbazole/H_2_O_2_. Histochemical staining for glucose-6-phosphatase activity was performed according to [36] on glutaradehyde-fixed slices.

### Western blotting

Whole cell extracts were denatured in Laemmli buffer at 40°C, separated by sodiumdodecylsulfate-polyacrylamide gel electrophoresis (SDS-PAGE; 50 μg of protein per lane) and transferred to PVDF membranes. Antibodies against E-cadherin (1:100; Becton Dickinson, Heidelberg, Germany; catalog no. 610181), OATP1A4 and OATP1A2 (see above; 1:200 dilution), and glyceraldehyde-3-phosphate dehydrogenase (1:1,000; Millipore, Chandler’s Ford, UK; catalog no. MAB374) were used in combination with alkaline phosphatase-conjugated secondary antibodies directed against mouse (1:10,000; Tropix, Weiterstadt, Germany; catalog no. AC32ML) or goat immunoglobulins (1:5,000; Santa Cruz Biotechnology; catalog no. sc-2022), with CDP-Star (Tropix) as a substrate. Chemiluminescence was monitored on a charge-coupled device (CCD) camera system (Raytest, Straubenhardt, Germany).

### Extraction of cholesterol and 4β-hydroxycholesterol

Serum cholesterol was determined by GC-MS as described previously [37] with minor modifications: briefly, 10 μl of serum were spiked with 10 μg of [^2^H_5_]-cholesterol as internal standard. After saponification with 0.5 ml 1 M NaOH in 90% ethanol at 70°C for 1 h, 250 μl H_2_O were added and the samples extracted with 2 ml n-hexane. A 50 μl aliquot of the extract was evaporated to dryness and derivatized with 20 μl *N,O*-bis(trimethylsilyl)trifluoroacetamide for 30 min at room temperature.

Sample preparation for 4β-hydroxycholesterol analysis was performed as previously published [38] with minor modifications. Briefly, liver tissue (10–30 mg) was homogenized in 400 μl phosphate buffer (0.1 M potassium phosphate, 2 mM EDTA, 11 μg/ml butylhydroxytoluene (BHT), pH 7.4) using a pestle. After addition of 10 μg BHT and 50 ng of the internal standard [^2^H_4_]-4β-hydroxycholesterol, the homogenate was saponified with 2.5 ml 1 M NaOH in 90% ethanol at 70°C for 1 h under argon and then extracted with 1 ml H_2_O and 5 ml CHCl_3_. The organic phase was evaporated to dryness in a stream of N_2_ and the residue dissolved in 1 ml toluene. Samples were purified by solid phase extraction on silica cartridges (Isolute Si 100 mg; Biotage, Uppsala, Sweden) preconditioned with n-hexane. Cartridges were washed with 1 ml n-hexane and 10 ml 2-propanol in n-hexane (0.5% v/v), and then eluted with 2 ml 2-propanol in n-hexane (30% v/v). The eluate was evaporated to dryness in a stream of N_2_ and derivatized as described above.

### Cholesterol and 4β-hydroxycholesterol quantification

A 5975C inert XL MSD in the EI mode, coupled to a 7890A gas chromatograph (GC; Agilent Technologies, Waldbronn, Germany) was used. GC was performed on a J&W DB-5MS column (25 m, 0.2 mm i.d., 0.33 μm film thickness; Agilent) in the splitless mode. For cholesterol analysis, the GC oven program started at 150°C and was held for 1 min. Temperature was increased with 20°C/min to 300°C, with a total run time of 18.5 min. The trimethylsilyl derivatives of cholesterol and the internal standard [^2^H_5_]-cholesterol were detected in SIM mode at m/z 458 and 463, respectively. For the analysis of 4β-hydroxycholesterol, the GC oven program started at 150°C and was held for 1 min. Temperature was increased with 10°C/min to 250°C, then with 30°C/min to 300°C. The trimethylsilyl derivatives of 4β-hydroxycholesterol and the internal standard [^2^H_4_]-4β-hydroxycholesterol were quantified in SIM mode at m/z 366 and 370, respectively, using m/z 456 and 460 as qualifier ions.

Calibration samples for cholesterol were prepared in isooctane with 10% 2-propanol in the concentration range from 1 μg to 30 μg. Calibration samples for 4β-hydroxycholesterol were prepared in isooctane with 0.8% 2-propanol from 5 ng to 100 ng.

Calibration samples were worked up as the samples, and analyzed together with the unknown samples. Calibration curves based on internal standard calibration were obtained by weighted (1/×) linear regression for the peak area ratio of the analyte to the respective internal standard against the amount of the analyte. The concentration in unknown samples was obtained from the regression line.

### Atorvastatin and 2-hydroxy-atorvastatin quantification

Tissue samples (50 to 150 mg) were homogenized in a total volume of 600 μl of acetate buffer (0.1 M, pH 5) and internal standard (100 pmol each of ^2^H_5_-atorvastatin and ^2^H_5_-2-hydroxyatorvastatin) in a FastPrep 24 homogenizer (MP Biomedicals, Santa Ana, USA) for 40s at speed 6.0 using lysing matrix D. The homogenate was extracted with ether:2-propanol 9:1 (v/v), and the organic phase evaporated in a stream of nitrogen. The residue was dissolved in aqueous ammonium acetate (10 mM with 1% formic acid):acetonitrile 60:40 (v/v) and determined by LC/MS-MS analysis as described [39].

### Quantification of tumor burden

Tumor burden was quantified as the area fraction (corresponding to the volume fraction) of glucose-6-phosphatase-altered lesions on an Axio Imager light microscope (Imager.M1; Zeiss, Göttingen, Germany). AxioVision software Rel.4.5 (Zeiss) was used to determine tumor and normal tissue areas. The calculation of the number and size of glucose-6-phosphatase lesions per cm^3^ of liver tissue was performed according to [40]. For each mouse, three liver lobes were examined (right lobe, left lateral lobe, and caudate lobe) using three slices per liver lobe with at least 20 sections distance between the individual slices. Hematoxylin/eosin staining of the tumors and immunohistochemical staining for glutamine synthetase and E-cadherin was assessed on parallel slices.

### Mutation analysis

Tumor tissue samples from mice of both treatment groups were punched out of liver slices (20 μm thickness) by a sharpened cannula. Following proteinase K digestion, genomic DNA was amplified by polymerase chain reaction (PCR) using the following primer pairs: Ha-ras_fwd 5′-GAGACATGTCTACTGGACATCTT-3′, Ha-ras_rev 5′-GCTAGCCATAGGTGGCTCACCTG-3′; B-raf_fwd 5′-TCAAAATGCTTTCTCTAATAGGA-3′, B-raf_rev 5′-TGTTCTGGAACTATATAGACAG-3′. PCR products were analyzed for mutations in Codon 61 of *Ha-ras* and Codon 637 of *B-raf* by restriction fragment length polymorphisms analysis as previously described [41].

### Cell culture and *in vitro*assays

Mouse hepatoma cell lines 53.2b, 55.1c, 70.4 and Hepa1c1c7 [42] were grown in D-MEM/F-12 medium (Invitrogen, Darmstadt, Germany) supplemented with 1% fetal bovine serum and antibiotics. For cytotoxicity testing, 9000 cells were seeded per well of a 96-well plate and treated with the indicated concentrations of atorvastatin for 24 h starting 24 h after seeding. Plates were analyzed using the neutral red uptake and Alamar Blue tests as previously described [43]. For the analysis of growth behavior, cells were seeded on 96 well-plates at a density of 5000 cells/well and treated with the indicated concentrations of atorvastatin starting 12 h after seeding. Solvent controls received medium containing 0.4% dimethyl sulfoxide. Medium was changed after 48 h. Following different incubation periods, cells were fixed by 10% trichloroacetid acid and analyzed photometrically after staining with sulforhodamine B according to [44].

### Gene expression analysis

Total RNA was isolated and reverse transcribed from cell cultures or liver tissue as previously described [42], using Trizol reagent (Invitrogen) and avian myeloblastosis virus reverse transcriptase (Promega, Mannheim, Germany). SYBR green I-based analyses of target gene expression were conducted on a LightCycler instrument (Roche) as previously described [42] using the following primer pairs: Hmgcr_fwd 5′-AGCAAGTGATTACCCTGAGTTTAG-3′, Hmgcr_rev 5′-CAGACATTCTTCATTAGGTCGTG-3′; Hmgcs1_fwd 5′-TTGGGGACGTTAAATTAGAAGAT-3′, Hmgcs1_rev 5′-CCAAGCCAGAACCGTAAGAG-3′; Oatp1a4_fwd 5′- GAAACAGTATTCCTCCACCATC-3′, Oatp1a4_rev 5′-TTGATAAGCCCAACTACAGACG-3′; Oatp1b2_fwd 5′-ATCCCGTGACTAATCCAACA-3′, Oatp1b2_rev 5′- ACCAAACTGCTGCTCTATAAACT-3′; 18S rRNA_fwd 5′-CGGCTACCACATCCAAGGAA-3′, 18S rRNA_rev 5′-GCTGGAATTACCGCGGCT-3′. The Mm_Cyp7a1_1_SG QuantiTect Primer Assay (Qiagen, Hilden, Germany) was used for Cyp7a1 determination. Expression was normalized to the housekeeping gene 18S rRNA using the Pfaffl method [45].

### Calculation of statistical significance

Statistical significance was calculated using an unpaired t-test with Welch’s correction. Homogeneity of variances was tested for using Bartlett’s test. The Shapiro-Wilk test was used to analyze normal distribution of values. Differences were considered significant when p < 0.05.

## Results

### Growth inhibition of murine tumor cell lines by atorvastatin *in vitro*

**Figure 1:**
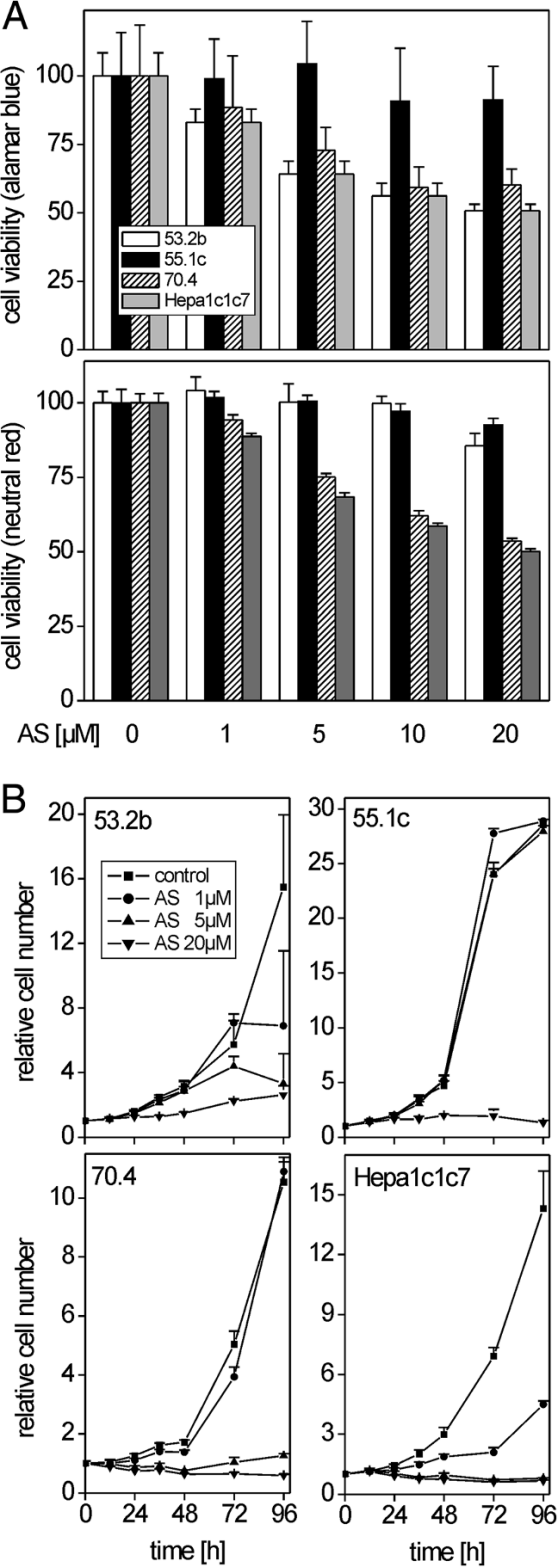
**Cytotoxicity and cell growth inhibition by atorvastatin** ***in vitro*** **. A**. Effects of different concentrations of atorvastatin on mouse hepatoma cells from lines 53.2b, 55.1c, 70.4 and Hepa1c1c7 after 24 h of treatment, as analyzed by the Alamar blue and neutral red uptake assays. **B**. Growth curves of cell cultures in the presence of atorvastatin. Representative data from 1 out of 4 experiments are shown as mean + SD of n = 6 technical replicates.

First, studies with mouse hepatoma cell lines were conducted to prove the *in vitro* efficacy of atorvastatin treatment of murine liver tumor cells. Mouse hepatoma cell lines 53.2b, 55.1c, 70.4, and Hepa1c1c7 were screened for their expression of *Hmgcs1* and *Hmgcr*, encoding the first and rate-limiting steps in cholesterol biosynthesis. All cell lines expressed the two mRNAs at levels comparable or slightly higher than normal mouse liver. *Cyp7a1* expression was not detectable (data not shown). A moderate therapeutic dose of 40 mg atorvastatin per day results in maximum human plasma levels of 66 ng/ml, corresponding to a plasma concentration of ~118 nM of the drug [46]. However, hepatic concentrations are ~50-fold higher than in plasma, as measured in atorvastatin-treated rats, resulting in an estimated liver concentration of ~6 μM [47]. Therefore, the ability of atorvastatin to interfere with hepatoma cell growth *in vitro* was tested at concentrations ranging from 1 μM to 20 μM, in order to meet the expected *in vivo* concentration of atorvastatin in mouse liver tumor cells. After 24 h of treatment, 55.1c cells appeared to be rather resistant to atorvastatin treatment up to the maximum concentration of 20 μM, while cells from lines 70.4 and Hepa1c1c7 showed the greatest sensitivity (Figure 1A). Long-term growth of atorvastatin-treated cells was monitored using the sulforhodamine B assay. Again, 55.1c cells were most resistant, with only the highest concentration of atorvastatin causing a remarkable inhibition of cell growth (Figure 1B). Hepa1c1c7 cells showed diminished growth already at 1 μM atorvastatin, while 53.2b cells were inhibited at concentrations ≥5 μM. In summary, atorvastatin was able to interfere with the viability and/or growth of different mouse hepatoma cell lines at *in vivo*-relevant concentrations.

### Effects of atorvastatin on hepatoma growth *in vivo*

**Figure 2:**
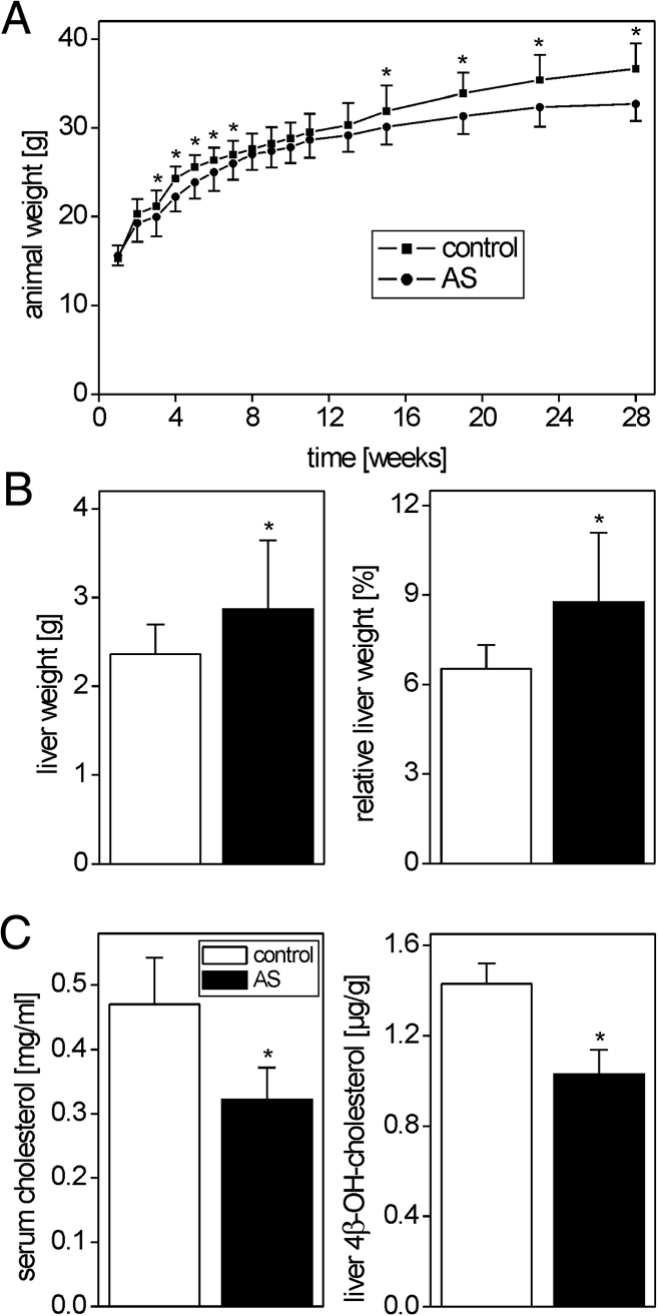
**Effects of atorvastatin (AS) treatment (0.1% in diet)** ***in vivo*** **. A**. Diminished weight gain in atorvastatin-treated mice. Mean + SD (n = 24-25) are shown. **B**. Increased liver weight and liver to body weight ratios in atorvastatin-treated mice. **C**. Reduction of plasma cholesterol and liver 4β-hydroxycholesterol levels. Mean + SD (n = 5; 5 randomly selected mice per group) are shown. Statistical significance (p < 0.05) is indicated by asterisks.

Chemically induced mouse liver tumors were generated by a single injection of DEN at 2 weeks of age according to [34]. Starting one week later, mice were stratified into a control (standard diet) and an atorvastatin group (0.1% atorvastatin in the diet). Atorvastatin feeding slightly but significantly inhibited weight gain of the animals in the atorvastatin group during the 6 months period of the experiment (Figure 2A). Atorvastatin-fed mice possessed larger livers and had an increased liver to body weight ratio (Figure 2B). In line with its expected pharmacological effects, atorvastatin significantly reduced serum cholesterol and liver 4β-hydroxycholesterol levels (Figure 2C). None of the animals died during the study or had to be sacrificed ahead of schedule.

**Figure 3:**
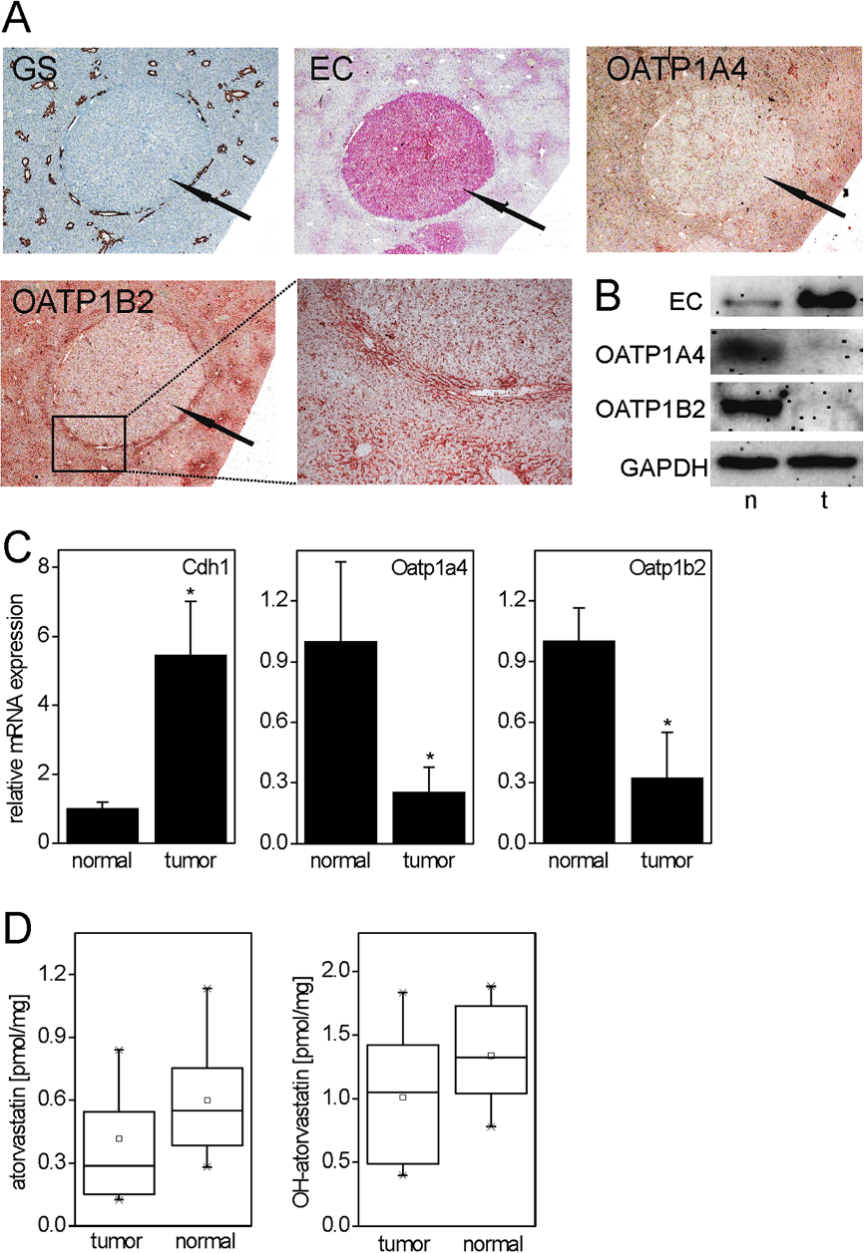
**Expression of the atorvastatin (AS) uptake transporters Oatp1a4 and Oatp1b2 in mouse liver tumors. A**. Diminished expression of OATP1A4 and OATP1B2 protein in tumor tissue, as detected by immunohistochemistry. For comparison, staining for glutamine synthetase (GS, not expressed in the tumors) and E-cadherin (EC, overexpressed in the tumors) is shown. **B**. Validation of protein expression results by Western blotting. Abbreviations: t, tumor; n, normal tissue. **C**. Diminished expression of Oatp1a4 (Slco1a4) and Oatp1b2 (Slco1b2) mRNAs in tumors (left diagrams). Mean + SD (n = 5-6) is shown. Statistical significance (p < 0.05) is indicated by asterisks. For comparison, expression of Cdh1 mRNA (encoding E-cadherin) is shown. Data from atorvastatin-treated livers are depicted; comparable results were obtained with untreated livers (not shown). **D**. Levels of atorvastatin and hydroxyatorvastatin in normal liver and liver tumors (n = 13-16).

At the end of the experiment, livers were isolated and the occurrence of hepatocellular tumors was analyzed. Three liver lobes were analyzed for each mouse (see Methods section). Tumor incidence was 100% in both groups and multiple tumors were detected per animal. This is in line with previous results from comparable experiments with DEN as a tumor inducer [32–34]. For a detailed list of the number of tumors analyzed per animal, please refer to the Additional file 1: Table S1. As also expected from previous studies, the vast majority (>95% by number and volume fraction) of the resulting tumors after 6 months were basophilic, E-cadherin-expressing and glutamine synthetase-deficient hepatocellular adenoma (data not shown; for representative immunostainings see Figure 3A). These characteristics are hallmarks of mouse liver adenoma with activated MAPK signaling; e.g. see [48]. Accordingly, mutation analyses revealed that 20 out of 26 analyzed tumors (77%) contained activating mutations in either Codon 61 of *Ha-ras* or Codon 637 of *B-raf* (Table 1), encoding key players in the MAPK cascade. Accordingly, *Ha-ras*-mutated tumors were strongly positive for the phosphorylated active MAPK downstream kinase ERK1/2 (data not shown).

**Table 1:** **Results of mutation analysis**

Mutation/AA exchange	No. of tumors
*Ha-ras* Cod. 61 Q ➔ R	7
*Ha-ras* Cod. 61 Q ➔ L	1
*Ha-ras* Cod. 61 Q ➔ K	4
*Ha-ras* Cod. 61 Q ➔ H	0
*B-raf* Cod. 637 V ➔ E	8
None detected	6

In total, 26 tumors were analyzed for mutations in *Ha-ras* or *B-raf*.

Note: The absence of *Ha-ras* or *B-raf* mutations in a tumor does not preclude activation of MAPK signaling in this tumor driven by other genetic alterations.

**Figure 4:**
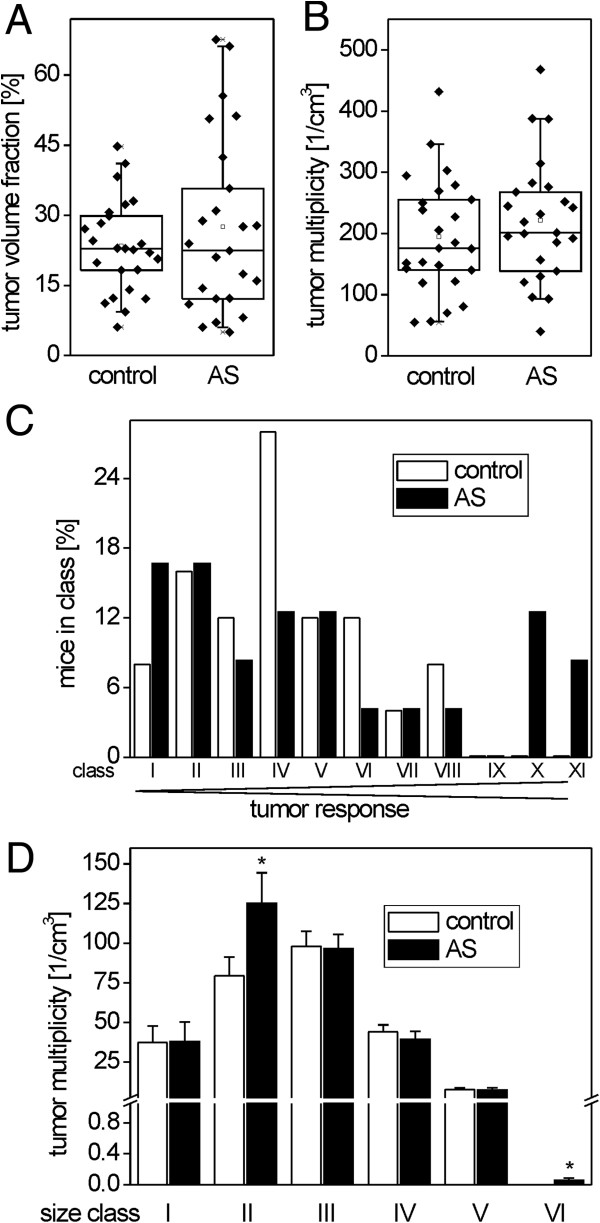
**Lack of atorvastatin effects on the growth of N-nitrosodiethylamine-induced mouse liver tumors. A**. Tumor volume fraction of control and atorvastatin-treated mice, as quantified by the analysis of glucose-6-phosphatase-altered lesion. **B**. Projected tumor multiplicity. Individual data from n = 24-25 mice per group are shown together with the corresponding box charts. **C**. Distribution of tumor volume fraction between mice from the two groups. Mice were grouped into the following responder classes according to their hepatic tumor volume fractions: I, tumor volume fraction <10%; II, 10-15%; III, 15.01-20%; IV, 20.01-25%; V, 25.01-30%; VI, 30.01-35%; VII, 35.01-40%; VIII, 40.01-45%; IX, 45.01-50%; X, 50.01-60%; XI, >60%. The atorvastatin group has a higher percentage of weak responders (class I), but also a higher percentage of strong responders (classes X and XI). **D**. Size class distribution of tumor multiplicity. Tumors were grouped into the following size classes: I, 0.05-0.25 mm diameter; II, 0.26-0.5 mm; III, 0.51-1.00 mm; IV, 1.01-2.00 mm; V, 2.01-6.5 mm; VI, >6.5 mm. Mean + SEM (n = 24-25) is shown. Statistical significance (p < 0.05) is indicated by asterisks. Very big tumors are exclusively observed in the atorvastatin group.

Quantification of the hepatic tumor volume fractions (Figure 4A) as well as the calculation of liver tumor multiplicities (Figure 4B) in the two groups revealed that no significant differences were present between atorvastatin-treated and control mice (for details please also refer to the Additional file 1: Table S1). However, variances of tumor volume fractions were not equal in the two groups, with the significantly higher variance in the atorvastatin group (p = 0.002; Bartlett’s test). Therefore, some differences were visible when mice were grouped into “responder classes”, according to their individual tumor burden (Figure 4C): with atorvastatin treatment, there were, on the one hand, comparably many weak responders with a hepatic tumor volume fraction of <15%. On the other hand, very strong responders with a hepatic tumor volume >50% were exclusively present in the atorvastatin-treated group. Similarly, tumors of the biggest size class, with a diameter of >6.5 mm, were observed in atorvastatin-treated livers only (Figure 4D). Tumor volume fraction values were normally distributed within the control group, but not in the atorvastatin group (p = 0.026; Shapiro-Wilk test). Altogether, this indicates a broader distribution of tumorigenic response in mice treated with atorvastatin, as compared to control mice. However, these effects of atorvastatin did not alter the overall mean tumor response of the population, which was not distinguishable from controls.

### Expression of atorvastatin uptake transporters and atorvastatin levels in tumors

Atorvastatin is taken up into hepatocytes by means of the organic anion transporters OATP1A4 and OATP1B2 [49]. To check whether inefficient uptake of atorvastatin might have caused the lack of efficacy of the drug on tumor development, the expression of these two transporters in normal liver and liver tumors was analyzed by immunohistochemistry, Western blotting, and real-time RT-PCR. The results are presented in Figure 3 and demonstrate a marked reduction of both transporters in liver tumor tissue, as compared to surrounding normal hepatocytes. Of note, OATP1B2 immunoreactivity was preferentially located in perivenous hepatocytes surrounding the central veins (Figure 3A). Our results are therefore in line with previous findings that mouse liver tumors with activated MAPK lack the expression of many ‘perivenous’ genes [48]. The down-regulation of the two transporters might indicate that the uptake of atorvastatin into tumor cells is reduced. Thus, the levels of atorvastatin and its major metabolite 2-hydroxyatorvastatin were analyzed by mass spectrometry in normal liver and tumor tissue (Figure 3D). Levels of both the drug and its metabolite were reduced in the tumors, but the effect slightly failed our criteria for statistical significance (p = 0.052 for atorvastatin and p = 0.068 for hydroxyatorvastatin, respectively).

## Discussion

Mouse liver tumors with an activated MAPK signaling pathway have been extensively characterized; they show high expression levels of cholesterol-synthesizing enzymes along with a down-regulation of the main cholesterol-metabolizing enzyme and possess elevated levels of cholesterol [32, 33]. Assuming that the metabolic pathways active in a certain type of tumor are beneficial for their growth, these tumors appear suited for a preventive approach based on a cholesterol-lowering compound such as atorvastatin. Possible beneficial effects of statin treatment regarding the formation of HCC are controversially discussed (see Background section). The present data conclusively show that DEN-induced tumor development in male C3H/HeN mice is not beneficially affected by treatment with atorvastatin. This is in line with the lack of tumor-inhibiting properties of atorvastatin in a mouse model of TSC2-related liver hemangioma [29], but not with data obtained with a MYC-driven HCC mouse model where atorvastatin acted as a tumor-preventive compound [25].

In principle, a lack of treatment efficacy might always be related to a too low dose of the respective agent. In the present study, 0.1% atorvastatin in the diet was able to slightly reduce the weight gain of the mice and to induce elevated liver weights, indicating that the maximum tolerated dose of the drug has been chosen for the experiment and that results obtained with higher dosing would always be questionable due to unspecific toxicity of the compound. Moreover, pharmacological efficacy of the drug was shown by a significant reduction of cholesterol levels in the treated population. The observed degree of serum cholesterol reduction is within the range of what is expected at therapeutic statin doses in humans [50]. Moreover, the *in vitro* experiments, where the different cell lines showed variable responses to atorvastatin treatment despite similar levels in cholesterol-synthesizing enzymes, suggest that effects other than HMGCR inhibition might contribute to the growth-inhibitory effect of the drug. Of note, it has been published that tumor-inhibitory effects of lovastatin even occur in the absence of measurable effects on cholesterol levels [28].

The daily food intake has not been measured during the present study. However, when calculating with published average values of a food intake of 5 g per mouse of 30 g body weight [51], the total oral uptake of atorvastatin in our experiment was approximately 167 mg/kg body weight/day. This is considerably higher than in the MYC-HCC experiment by [25], where the drug had been administered three times per week at 100 mg/kg body weight, which led to an inhibition of tumor growth. In synopsis, all these parameters indicate that the selected atorvastatin dose was not too low to produce relevant effects. The differences between our results and the previous study with MYC-induced HCCs [25] might result from the differences between the physiology of transgenic MYC-driven HCCs and our chemically induced tumors. Differences between mouse strains might also play a role in the response to atorvastatin treatment. Unfortunately, no information about the genetic background of the mice is provided in [25]. Moreover, the low number of only 5 animals per group in the previous study [25] hampers statistical evaluation and interpretation of these results.

In humans, atorvastatin is mainly metabolized by cytochrome (CYP) P450 3A4 [52]. Accelerated metabolism of the drug in the tumor cells might also be responsible for the lack of treatment efficacy in our experimental system. Mouse liver tumors with an activated MAPK pathway show de-regulated, mostly diminished, mRNA expression of various CYPs. Enzymes from the Cyp3a family, however, are not significantly altered in MAPK-activated mouse liver tumors, as compared to normal tissue [32, 33]. This indicates that major alterations in the metabolism of atorvastatin are not to be expected in the tumor cells.

An inefficient uptake of the drug into the tumor cells might explain also the lack of atorvastatin efficacy in our experiment. We show that organic anion transporters involved in the uptake of atorvastatin, namely Oatp1b2 (Slco1b2) and Oatp1a4 (Slco1a4) [49], are strongly down-regulated in the tumors at the mRNA and protein levels. Studies with human HCC samples have revealed the down-regulation of OATP1B1 [53, 54], the human atorvastatin-transporting protein [49]. In view of this fact, it is tempting to speculate that human hepatocellular tumors might exhibit a reduced ability to take up atorvastatin and probably also other structurally related statins. This scenario implies that statins might not be effective in tumor cells when administered at the therapeutic dose, thus arguing against the proposed protective effect of statins, given the fact that the putative tumor-inhibiting properties of statins are based on direct effects of the drug on tumors cells, not on indirect effects involving statin effects on tumor-surrounding non-tumorous cells. However, the observed reduction of atorvastatin levels in mouse liver tumors is not very pronounced. Therefore, it seems rather unlikely that the apparent lack of tumor inhibition in our experiment can be solely explained by a diminished uptake of the drug into the tumors.

In summary, present data challenge the idea that atorvastatin inhibits tumor development in the liver. Nonetheless, it has to be noted that results from animal experiments cannot be transferred to the situation in humans with absolute certainty, in this particular case for example due to possible species differences in tumor genetics and biology, and/or due to the much higher interindividual variability in the human population, as compared to inbred mouse strains. However, in the absence of unequivocal epidemiological data, results from animal experimentation are an important and indispensable source of information.

## Conclusions

The present data provide substantial evidence that atorvastatin does not beneficially influence tumor growth in mouse liver and thereby challenge the hypothesis that statin use might protect against hepatocellular cancer.

## Electronic supplementary material

Additional file 1: Table S1: Supplementary details of tumor analysis and quantification. (PDF 63 KB)

## Abbreviations

BHT: Butylated hydroxytoluene
CCD: Charge-coupled device
CYP: Cytochrome P450
DEN: Diethylnitrosamine
GC: Gas chromatograph
HCC: Hepatocellular carcinoma
*Hmgcs*: 3-hydroxy-3-methylglutaryl-CoA synthase
Hmgcr: 3-hydroxy-3-methyl-glutaryl-CoA reductase
MAPK: Mitogen-activated protein kinase
Lss: Lanosterol synthase
OATP: Organic anion transporting peptide
(RT)-PCR: (Real time) polymerase chain reaction
SDS-PAGE: Sodiumdodecylsulfate-polyacrylaminde gel electrophoresis.
