# Supplementary material for: Chemically induced mouse liver tumors are resistant to treatment with atorvastatin
Source: BMC Cancer. 2014 Oct 15;14:766. doi: 10.1186/1471-2407-14-766 (PMC4203962; doi:10.1186/1471-2407-14-766)
Supplement: Supplementary file 1 — Additional file 1: Table S1: Supplementary details of tumor analysis and quantification. (PDF 63 KB) [file 12885_2014_4942_MOESM1_ESM.pdf]

tumor area (glucose-6-phosphatase-altered lesions)

internal animal ID	treatment	tumors detected (sum of lobes 1-3)	mean tumor diameter [cm]	tumor number [1/cm3]	tumor volume fraction [%]
1	control	32	0.1059	147.93	32.3147
2	control	31	0.0807	176.07	9.3382
7	control	22	0.1398	70.17	22.8666
8	control	12	0.1144	56.33	6.1051
12	control	48	0.1183	153.15	22.9514
13	control	36	0.1257	119.41	18.2345
41	control	32	0.1142	121.62	12.2960
42	control	41	0.1098	185.15	22.6045
29	control	43	0.1035	151.35	14.0920
30	control	62	0.0957	269.31	29.8467
35	control	52	0.1064	205.53	23.8751
60	control	31	0.0644	250.12	11.1954
69	control	16	0.1254	80.67	18.3224
72	control	19	0.1727	54.70	28.2829
63	control	27	0.0996	142.78	22.0240
65	control	59	0.1014	302.80	19.8669
38	control	70	0.1038	278.78	41.0664
56	control	51	0.0987	238.53	24.5443
18	control	33	0.0970	140.14	12.1537
19	control	46	0.0824	294.52	20.6544
24	control	57	0.1100	255.21	38.2776
25	control	64	0.1006	227.21	27.0908
47	control	61	0.0822	431.48	33.0171
50	control	62	0.0717	345.87	30.6179
51	control	67	0.1227	175.11	44.7401
33	atorvastatin	56	0.0974	231.78	35.7391
34	atorvastatin	60	0.0759	387.04	22.4579
5	atorvastatin	31	0.0789	156.77	12.2452
6	atorvastatin	23	0.0421	314.11	5.0006
11	atorvastatin	18	0.1022	93.20	7.0922
16	atorvastatin	22	0.1663	129.78	66.1469
40	atorvastatin	41	0.1094	95.71	67.5854
45	atorvastatin	44	0.0864	199.68	12.1372
46	atorvastatin	50	0.0775	276.11	21.0360
49	atorvastatin	45	0.1215	185.42	55.5227
54	atorvastatin	53	0.1102	218.63	30.9578
17	atorvastatin	52	0.1035	195.27	27.5594
37	atorvastatin	73	0.1084	192.03	50.6564
55	atorvastatin	83	0.0767	387.75	17.4308
39	atorvastatin	74	0.0947	282.43	28.8013
59	atorvastatin	43	0.0996	120.50	42.3810
22	atorvastatin	27	0.0802	201.28	14.3876
23	atorvastatin	50	0.1052	39.70	15.9980
28	atorvastatin	50	0.0781	244.50	11.0061
62	atorvastatin	49	0.0983	242.33	27.8033
71	atorvastatin	29	0.0627	251.80	8.1210
74	atorvastatin	68	0.0812	467.79	23.9348
64	atorvastatin	34	0.1391	138.57	51.2655
68	atorvastatin	29	0.0616	267.47	6.0529
